# Linking brain electrical signals elicited by current outcomes with future risk decision-making

**DOI:** 10.3389/fnbeh.2014.00084

**Published:** 2014-03-18

**Authors:** Dandan Zhang, Ruolei Gu, Lucas S. Broster, Yang Jiang, Wenbo Luo, Jian Zhang, Yue-jia Luo

**Affiliations:** ^1^Institute of Affective and Social Neuroscience, School of Medicine, Shenzhen UniversityShenzhen, China; ^2^Key Laboratory of Behavioral Science, Institute of Psychology, Chinese Academy of SciencesBeijing, China; ^3^Department of Behavioral Science, University of Kentucky College of MedicineLexington, KY, USA; ^4^Department of Psychology, School of Psychology, Liaoning Normal UniversityDalian, China

**Keywords:** decision-making, outcome evaluation, event-related potential, event-related oscillation, time-frequency analysis, independent component analysis

## Abstract

The experience of current outcomes influences future decisions in various ways. The neural mechanism of this phenomenon may help to clarify the determinants of decision-making. In this study, thirty-nine young adults finished a risky gambling task by choosing between a high- and a low-risk option in each trial during electroencephalographic data collection. We found that risk-taking strategies significantly modulated mean amplitudes of the event-related potential (ERP) component P3, particularly at the central scalp. The event-related spectral perturbation and the inter-trial coherence measurements of the independent component analysis (ICA) data indicated that the “stay” vs. “switch” electrophysiological difference associated with subsequent decision-making was mainly due to fronto-central theta and left/right mu independent components. Event-related cross-coherence results suggested that the neural information of action monitoring and updating emerged in the fronto-central cortex and propagated to sensorimotor area for further behavior adjustment. Based on these findings of ERP and event-related oscillation (ERO) measures, we propose a neural model of the influence of current outcomes on future decisions.

## Introduction

Decision-making, which refers to the process of making choices among various options, can be temporally divided into partially distinct phases, including the assessment of available options, the execution of an action, and the evaluation of outcome feedback (Fellows, 2004; Paulus, 2005; Rangel et al., 2008). Importantly, outcome evaluation should not be regarded as the ending of this process. Rather, the outcome information is stored in memory to help exploring action-outcome association (i.e., learning), so as to facilitate decision-making in similar occasions (Platt, 2002; Ernst and Paulus, 2005; Kahnt et al., 2009). In addition, current outcomes may affect future behavior by modulating the decision-maker's motivational states, such that rewards and punishments give arise to the tendencies of approach and avoidance, respectively (Schultz, 2004). Numerous economic and cognitive research have demonstrated that current outcomes strongly influence following decisions on a trial-by-trial basis, but the brain mechanisms underlying this kind of behavioral adjustment are largely unknown. Identifying a link between the neural activities elicited by current outcomes and subsequent decision strategies leads to a better understanding of the determinants of decision-making (Cohen et al., 2011; Wunderlich et al., 2011).

Event-related potentials (ERPs), which are based on electroencephalography (EEG) with exquisite temporal resolution, are well-suited to investigate the dynamic mechanisms of cognitive processes (Amodio et al., 2013). Investigating the potential associations between ERP signals elicited by current outcomes and subsequent behavioral decisions has important implications on how current outcomes shape future actions (Cohen et al., 2011). Two ERP components, namely feedback-related negativity (FRN) and the P3, are considered to be the major biomarkers of outcome processing (Gehring and Willoughby, 2002; Yeung and Sanfey, 2004; Philiastides et al., 2010; Walsh and Anderson, 2012). It has been widely suggested that FRN represents a signal of reward prediction error that mediates feedback learning and adaptive modification of behavior (Holroyd and Coles, 2002; Cohen et al., 2011; Walsh and Anderson, 2011). Cohen and Ranganath (2007) asked participants to play a strategic economic game against a computer opponent, and they discovered that FRN magnitude after losses predicted whether participants would change decision behavior on subsequent trials (see also Cavanagh et al., 2010; but see Chase et al., 2011). In contrast, San Martín et al. (2013) argued that the P3, which is associated with a memory updating process that guides future behavior, predicted decision adjustment on subsequent trials. In a probabilistic gambling task which required players to learn the optimal strategy, San Martín et al. (2013) found out that a larger fronto-central P3a indicated higher likelihoods for the participants to change their choices, while the FRN showed no relation with behavioral data (see also Chase et al., 2011; Ernst and Steinhauser, 2012; Zhang et al., 2013). In sum, previous studies have yielded heterogeneous findings about the relationship between the ERP components following outcome presentation and subsequent decision-making behavior. Thus, the electrophysiological mechanisms of the impact of current outcomes on future behavior remain unclear.

In our opinion, a few issues related to the research on this topic need to be addressed. First, the factor of task design should be carefully considered. Many previous studies used probabilistic learning tasks, in which positive outcomes appear more frequently than negative outcomes as participants successfully learn the winning rules (e.g., Bellebaum and Daum, 2008; Cavanagh et al., 2010; San Martín et al., 2013). Thus, in these studies, the changes of ERP amplitudes might be interpreted in terms of outcome probability rather than behavioral adjustment, since both the FRN and the P3 are sensitive to event probability (Polich and Criado, 2006; San Martín, 2012). Importantly, our previous studies have demonstrated that even though the probabilities of winning and losing were set to be equal, the participants still tried their best to explore an optimal strategy indicated by task instructions, and their behavioral decisions significantly deviated from chance level (Gu et al., 2010a,b; Zhang et al., 2013; see Hake and Hyman, 1953 for detailed discussion). Accordingly, we continued to set the probabilities of win and loss as equivalent in the current study (see Gehring and Willoughby, 2002, for further explanations).

Second, regarding that the electrophysiological activity of the brain is strongly oscillatory, a large amount of cognitively relevant EEG information is lost in time-locked averaging (Cohen, 2011). To overcome this shortcoming, event-related oscillations (EROs) may be used to capture cognitive processes that could not be reflected by traditional ERPs (Buzsaki and Draguhn, 2004; Makeig et al., 2004; Onton and Makeig, 2006; Knyazev, 2007). The attempt to explore cognitive dynamics during the decision-making process with EROs has revealed to be fruitful (Cohen et al., 2011). Accordingly, the current study also investigated the validity of ERO measures as behavioral predictors. Numerous studies have been devoted to this issue (e.g., Cohen et al., 2007; for a review, see Cohen et al., 2011), yet the comprehensive examination and direct comparison of the predictive powers of ERP and ERO indexes are rare.

Finally, many neuroscience studies have employed EEG or functional magnetic resonance imaging (fMRI) measures to investigate the relation between brain activity and decision-making (e.g., Knutson et al., 2007; San Martín et al., 2013). However, to the best of our knowledge, a prediction of future decisions on the single-trial level is still absent. In our opinion, a successful single-trial behavioral prediction would help to demonstrate the reliability of electrophysiological biomarkers associated with decision-making. Therefore in the current study, single-trial analysis was conducted based on both the ERP and ERO measurements.

The present study focused on the relationship between cortical electrical signals following current outcome presentation and subsequent behavioral output in a risk decision-making scenario. We employed EEG recording in a trial-by-trial gambling task to investigate the ERPs (specifically, the FRN and P3 components) and EROs. The predictive powers of these measures on risk-taking decisions were examined using single-trial analysis.

## Materials and methods

### Participants

Thirty-nine Chinese students (18 females; mean age 20.47 ± 2.26 years) were recruited from Beijing Normal University as paid volunteers. All participants were free of regular use of medication or other non-medical substances that could potentially affect the central nervous system. All were right-handed and had normal vision (with correction). All participants gave their written informed consents prior to the experiment. The experimental protocol was approved by the local Ethics Committee (Beijing Normal University).

### Behavioral procedure

To investigate the impact of current outcomes on subsequent decisions, this study employed the trial-by-trial paradigm in which the outcome presentation of one trial is immediately followed by the choice period of the next trial (Hertwig and Erev, 2009; Peterson et al., 2011). Before the task, participants were told that they would be involved in a monetary gambling game. They were informed about the rules and the meanings of symbols in the task and were asked to respond in a way that would maximize the total score amount. The higher the score they earned, the more bonus money they would receive at the end of the experiment.

**Figure 1:**
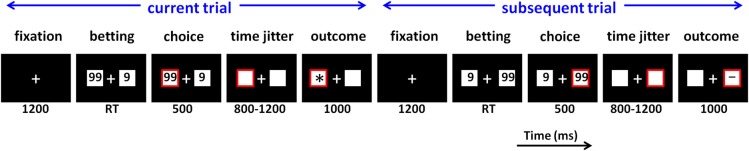
**Schematic diagram of two neighboring experimental trials in the monetary gambling task**. RT, response time. In this example, the participant chooses “99” in both the current and subsequent trials, which means he/she decides to stay in the “99” option in the subsequent trial rather than to switch to the “9” option.

During the formal task, participants sat comfortably in an electrically-shielded room approximately 100 cm in front of a computer screen. Experimental procedure is illustrated in Figure 1. Each trial began with the presentation of a central fixation point (white against a black background). After 1200 ms, two white rectangles (2.5° × 2.5° of visual angle) appeared on the left and right sides of the fixation point, displaying the numbers “9” and “99” (indicating the gambling points). Regarding their magnitude, the “9” was the low-risk option (low return and small loss) while the “99” was the high-risk option. The left-/right-ness of these two numbers were counterbalanced across the trials. Participants gambled by selecting the option displayed in the left or right rectangle by pressing the “F” or “J” button on a conventional computer keyboard with their left or right index finger. The chosen rectangle was highlighted by a red outline for 500 ms, followed by a time jitter between 800 and 1200 ms. Finally, the outcome of the participants' choice was presented in the chosen rectangle for 1000 ms.

In order to produce a highly dynamic decision-making scenario (Sengupta and Abdel-Hamid, 1993), various kinds of outcome valence were provided, including positive (“+”), negative (“−”), neutral (“0”), and ambiguous (“^*^”). The positive valence indicated that participants won the points that were chosen in this trial, while negative valence indicated they lost the points. The neutral valence meant participants neither won nor loss. The ambiguous outcome was uninformative, of which the valence could be positive, negative, or neutral (Holroyd et al., 2006; Bach and Dolan, 2012). Detailed instructions on the meanings of the symbols are described in Appendix (Part A).

The formal task consisted of four blocks of 160 trials each (640 trials in total). Blocks were separated by self-terminated breaks. Stimulus display and behavioral data acquisition were conducted using E-Prime software (Version 1.1, Psychology Software Tools, Inc., Pittsburgh, PA). Unbeknownst to the participants, the occurrences of four kinds of outcome valence were equiprobable regardless of participants' task performance.

The current study focused on the factor of subsequent strategy, which denotes the selection in the following trials (stay vs. switch); “stay” means the same option being chosen in the current trial and the next trial while “switch” means different options being chosen in two neighboring trials (see Figure 1). We characterized the *subsequent strategy* factor in terms of switch and stay not only because of previous research (Daw et al., 2006; Cohen and Ranganath, 2007; Boorman et al., 2009; San Martín et al., 2013), but also because one of our recent studies using a similar task design revealed that the stay/switch classification better accounted for the association between ERP results and future behavior than the high-risk/low-risk classification (Zhang et al., 2013).

**Figure 2:**
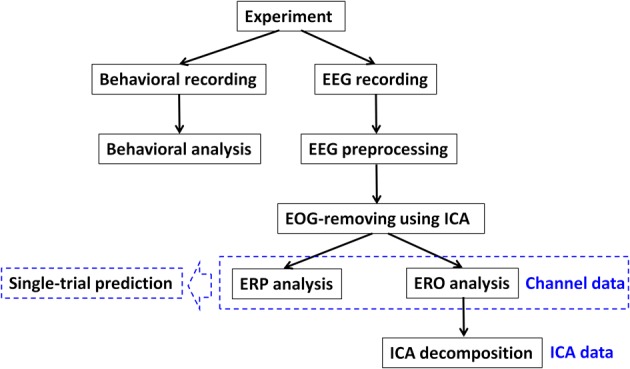
**The procedure of data recording and analysis in this study**.

Procedures of data recording and analysis of this study are illustrated in Figure 2. The statistical methods for behavioral and ERP data are described in Appendix (Part B).

### Behavioral measures

The “9” is defined as the low-risk option while the “99” is defined as the high-risk option. The tendency to choose the low-risk option indicates a preference for a risk-avoidant strategy. This preference was measured as the “risk-avoidant ratio,” by dividing the number of risk-avoidant choices by the total number of choices.

As described above, the current study was interested in whether participants switched their decision-making strategy in adjacent trials. The frequency of “choice-switching,” whether from the low-risk to high-risk option or the reverse, was measured as the “switch ratio,” by dividing the number of switched choices by the total number of choices.

### EEG recording and preprocessing

Electroencephalogram activity was recorded from 64 scalp sites using tin electrodes mounted in an elastic cap (NeuroScan Inc., Herndon, USA), with an online reference to the left mastoid and off-line re-referencing to the average of the left and right mastoids. Besides two electrooculogram (EOG) channels and the referential electrode at the right mastoid, 61-channel EEG data were collected with impedance levels kept below 5 kΩ. The electrode locations are shown in Appendix (Part G). EEG signals were continuously sampled at 500 Hz and filtered within 0.05–100 Hz.

EEG data processing was performed using a self-coded Matlab program based on the signal processing toolbox of Matlab R2011a (MathWorks, Natick, USA). The recorded EEG data were down-sampled to 250 Hz and further band-pass filtered (0.5–35 Hz) using a phase-shift free Butterworth filter (12 dB/Octave). The relatively high cutoff frequency for high-pass filtering was necessary to remove linear trends in the EEG, so as to ensure a reliable result of independent component analysis (ICA) process (Jung et al., 2005; Delorme et al., 2012). Filtered data were segmented beginning 1 s prior to the onset of outcome presentation with 3 s segments. The extended length of the epochs was necessary to perform time-frequency analyses with a high resolution.

### ICA procedure

Measuring non-phase-locked modulations in EEG spectral power (i.e., the spectral power of EROs) can be accomplished by jointly using time and frequency information. However, due to volume conduction, the scalp EEG represents a spatially-mixed signature of brain activity from different neural sources. Temporal separation of EEG data by means of ICA provides a more functionally-relevant analysis of brain dynamics and allows investigating characteristic time and spatial signatures of the distinct sources underlying the recorded channel data (Milne et al., 2009).

As shown in Figure 2, the EEG data were first separated by ICA into a sum of temporally-independent, spatially-fixed components arising from brain or extra-brain sources, followed by the analyses of noise-IC-removed channel data and ICA data. ICA was performed using EEGLAB 11.0.2.1 b, a freely available Matlab toolbox developed by Delorme and Makeig (2004). Detailed noise rejection and ICA procedure is described in Appendix (Part C). Finally, there were 105 ± 44 epochs being rejected per dataset after ICA. In particular, each participant had 211 ± 60 “switch” trials (range = 108–303) and 321 ± 64 “stay” trials (range = 149–421) for further analyses.

### Channel data analyses

ICs accounting for blinks and lateral eye movements were visually identified according to their scalp maps, component activations, and power spectra. These ICs were removed from each dataset; the remaining ICs were back-projected to reconstruct channel EEG data without EOG.

All epochs were baseline-corrected with respect to the mean voltage over the 200 ms preceding the onset of outcome presentation, followed by averaging in association with (a) the four outcome valences in the current trial and (b) the switch or stay strategy in the subsequent trial. Two ERP components (the FRN and P3) were analyzed based on the conventionally-averaged ERPs. The amplitude of the FRN was measured as the peak-to-peak difference between the most negative peak in the 200–280 ms window and the average voltage of the immediately preceding and following positive peaks, so as to eliminate the potential influence of other ERP components that temporally overlapped the FRN (Yeung and Sanfey, 2004; Chase et al., 2011). The P3 was measured as the mean voltage within the 320–500 ms time window.

Trial-by-trial stimulus-induced spectral power modulation and event-locked phase concentration of the EEG rhythms were studied using the event-related spectral perturbation (ERSP) and the inter-trial coherence (ITC) measurements in EEGLAB (Appendix, Part D).

### ICA data analyses

In total, the ICA algorithm produced 2379 ICs from 39 datasets (39 datasets × 61 ICs). To remove the ICs representing artifacts or other non-brain physiological sources, an equivalent current dipole model for each IC scalp topography was estimated using DIPFIT 2.2 (as EEGLAB plug-in)based on a four-shell spherical head model. Since it has been demonstrated that the EEG sources have scalp maps that nearly perfectly match the projection of a single equivalent brain dipole (Delorme and Makeig, 2004; Delorme et al., 2012), ICs with equivalent dipoles which computed projection to the scalp electrodes accounted for less than 95% of actual IC scalp map variance were not further analyzed. ICs with equivalent dipole located outside of the model head sphere were also removed. This exclusion procedure resulted an average of 10 brain activity ICs per subject (range = 6–15, total in 39 participants = 380).

Unlike univariate methods such as the general linear model, ICA is not naturally suited to generalize results from a group of subjects (i.e., ICA is a subject-based method and may sometimes produce ICs with different psychophysiological significances between subjects) (Esposito et al., 2005; Onton et al., 2006). To summarize results of ICA-based analysis across individuals, EEGLAB combines ICs from different subjects with clustering techniques (refer to Appendix, Part E for the IC cluster procedure). IC clusters of interest were further analyzed using mean scalp maps, power spectra, equivalent dipole locations, ERSP, ITC, and event-related cross-coherence (ERCOH) measures (Appendix, Part F).

### Single-trial prediction

Finally, the prediction power of single-trial channel data was investigated based on ERP and ERO features. To obtain a robust measurement of the spatiotemporal information buried in switch and stay trials, we extracted the baseline-corrected activity averaged across two time intervals, i.e., 200–280 and 320–500 ms, at all 61 channels as ERP features (Philiastides et al., 2006; Steinhauser and Yeung, 2010; Blankertz et al., 2011). For the ERSP feature, the baseline-normalized log spectral power was averaged within the time-frequency region of interest (hereafter referred to as TF ROI) of 200 to 500 ms × 3 to 7 Hz (see the blue box in Figure 4) at 61 channels. The TF ROI measurement of induced EEG activity has been employed by other researchers (e.g., Schulz et al., 2012). Since there was no significant ITC difference between conditions, the time-frequency feature was only extracted from ERSPs. The resultant 3 × 61 feature matrix was stacked into a feature vector of 183 dimensions by concatenating the ERP and ERSP measurements in 61 channels. Finally, PCA was employed to shrink the feature dimension to 10, since a large dimension (i.e., 183) of classification features would likely lead to a poor predictive performance due to overfitting (Blankertz et al., 2011).

The 10-dimensional feature vector was put into logistic regression classifiers (Parra et al., 2002; Philiastides et al., 2010; Steinhauser and Yeung, 2010) to label each trial as “switch” or “stay.” To evaluate the performance of the classifiers, the receiver operating characteristic (ROC) curve was plotted and the area under the curve (AUC) was calculated to quantify the categorization results (Parra et al., 2002; Philiastides et al., 2006, 2010; Steinhauser and Yeung, 2010). A 10-fold cross-validation was utilized to provide an unbiased evaluation of the classifier's performance in each participant (Parra et al., 2002; Philiastides et al., 2006, 2010; Pessoa and Padmala, 2007; Steinhauser and Yeung, 2010). Finally, a permutation statistical analysis was performed to test whether the achieved AUC values exceeded chance (Philiastides et al., 2006, 2010; Pessoa and Padmala, 2007; Steinhauser and Yeung, 2010; Schulz et al., 2012). The permutation procedure was applied to calculate the 99% confidence interval (CI) of the AUC with label-permuted trials (repeated 5000 times) to produce a distribution of AUC under the null hypothesis (i.e., the classifier has no discriminant ability). We then checked whether the resultant AUC given by classifiers was outside of the 99% CI of the associated label-permuted distribution, in which case we determined that the AUC achieved a significance level of *p* < 0.01.

## Results

### Behavioral results

The average risk-avoidant ratio was 49.6 ± 15.2% (mean ± *SD*) in 640 trials, which showed no difference compared with chance (50%) according to the one-sample *t* test (*p* = 0.86). The average switch ratio was 39.5 ± 11.2% in 636 trials (the last trial of each block was removed), which was significantly less than chance [*t*_(38)_ = −5.89, *p* < 0.001]; participants were more likely to repeat the same strategy than to switch strategies between neighboring trials.

A repeated-measures single-factor ANOVA was performed with outcome valence as the within-subject factor and with the switch ratio as the dependent variable, followed by pairwise comparisons. The switch ratio was significantly affected by outcome valence [*F*_(3, 114)_ = 41.9, *p* < 0.001, η^2^_*p*_ = 0.374]; it was larger following positive outcomes (49.4 ± 14.9%) than following the other three outcome valences (negative: 36.4 ± 12.4%, neutral: 35.0 ± 13.2%, ambiguous: 37.0 ± 13.3%; *ps* < 0.001).

### Channel data results

#### ERPs

**Figure 3:**
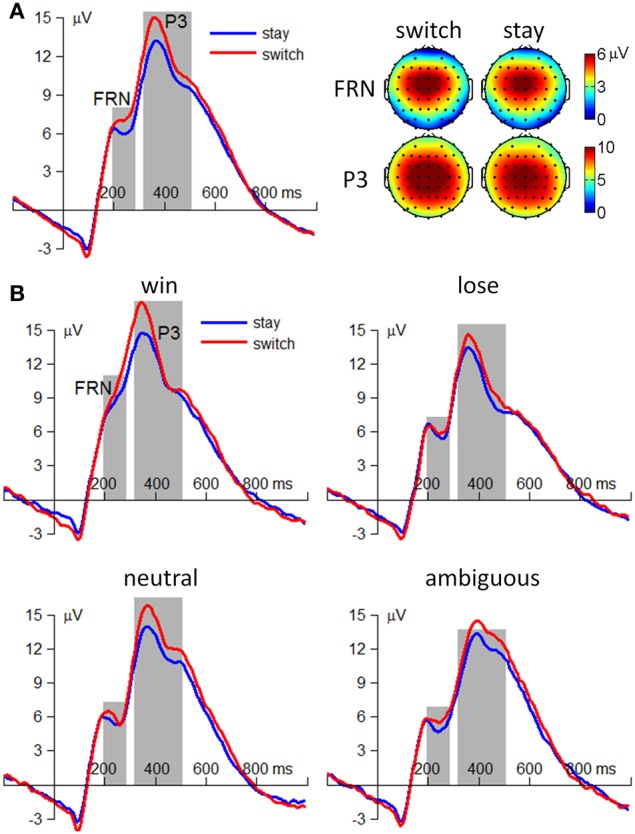
**ERP results from 39 participants**. **(A)** The main effect of subsequent strategy. Left: grand-mean ERP waveforms at electrode site Cz. Right: scalp topographies averaged from 200 to 280 ms for the FRN and from 320 to 500 ms for the P3. **(B)** The interaction effect of outcome valence by subsequent strategy. The *t* = 0 ms indicated the onset of outcome presentation.

The amplitude of the FRN was most prominent at the fronto-central area and was measured as the average potential at electrode sites FCz, FC1, FC2, Cz, C1, and C2 (see Figure 3A). A repeated-measures 4 (outcome valence: positive, negative, neutral, and ambiguous) × 2 (subsequent strategy: switch and stay) ANOVA was performed on the FRN amplitude. The main effect of outcome valence [*F*_(3, 114)_ = 47.8, *p* < 0.001, η^2^_*p*_ = 0.557] was significant; the FRN was smaller following positive outcomes [−4.6 ± 3.4μV] than following the other three conditions (negative: −7.6 ± 3.9μV, neutral: −7.3 ± 3.8μV, ambiguous: −7.2 ± 3.1μV; *ps* < 0.001). The main effect of subsequent strategy [*F*_(1, 38)_ = 3.35, *p* = 0.075, η^2^_*p*_ = 0.081] was not significant (switch = −6.8 ± 4.0μV; stay = −6.6 ± 3.5μV). The interaction effect of outcome valence by subsequent strategy was not significant [*F*_(3, 114)_ < 1, *p* > 0.05; see Figure 3B].

The amplitude of the P3 was most prominent at the central area and was measured as the average potentials at electrode sites FCz, FC1, FC2, Cz, C1, C2, CPz, CP1, and CP2 (see Figure 3A). Similar with the FRN, a repeated-measures 4 × 2 ANOVA was performed on the P3 amplitude. The main effects of outcome valence [*F*_(3, 114)_ = 9.82, *p* < 0.001, η^2^_*p*_ = 0.205] and subsequent strategy [*F*_(1, 38)_ = 18.4, *p* < 0.001, η^2^_*p*_ = 0.326] were significant. Regarding outcome valence, the P3 was larger following positive and neutral outcomes (positive: 13 ± 5.1 μV, neutral: 13 ± 5.3 μV) than following negative and ambiguous outcomes (negative: 11 ± 5.9 μV; ambiguous: 11 ± 3.9 μV; *ps* = 0.001–0.010). Regarding subsequent strategy, the outcome-elicited P3 amplitude was larger in trials followed by a switch strategy (13 ± 5.4 μV) than those followed by a stay strategy (11 ± 4.8 μV; see Figure 3A). The interaction effect of outcome valence by subsequent strategy was not significant [*F*_(3, 114)_ < 1, *p* > 0.05; see Figure 3B].

#### EROs

**Figure 4:**
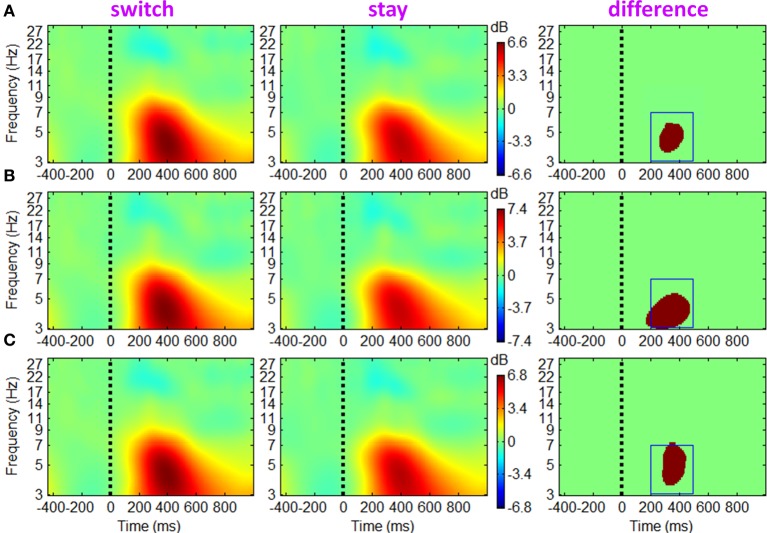
**Grand-mean ERSPs of the main effect of subsequent strategy based on channel EEG data**. Results obtained from sites Fz, Cz and Pz are shown in **(A–C)**. Colored ERSP images of switch and stay trials (the subplots in the first two columns) were produced using permutation statistics, with the red and blue indicating power increase or decrease; green areas indicate non-significance (*p* > 0.01). Binary images (i.e., the subplots in the third column) show significant ERSP differences between the two conditions based on a paired *t*-test with a significance threshold of *p* < 0.001; green areas indicate non-significance (*p* > 0.001). The blue box defines a time-frequency region of interest (TF ROI) for single-trial feature extraction.

**Figure 5:**
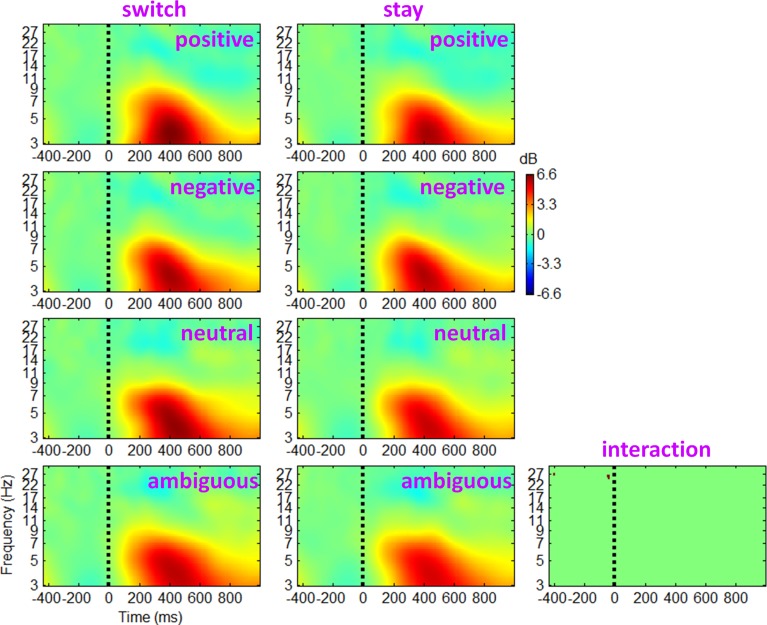
**Grand-mean ERSPs of the interaction effect of outcome valence by subsequent strategy based on channel EEG data**. Results obtained from the electrode site Cz. Colored ERSP images were produced using permutation statistics, with red and blue indicating power increase or decrease, respectively. The binary image shows a significant interaction effect; green areas indicate non-significance.

**Figure A1:**
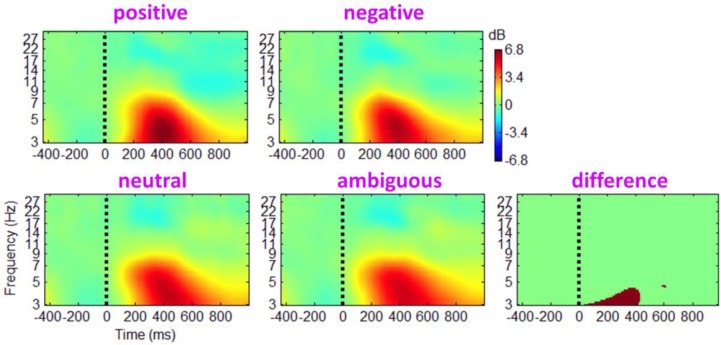
**Grand-mean ERSPs of the main effect of outcome valence based on channel EEG data**. Results obtained from the electrode site Cz.

The ERSP and ITC measurements for trials followed by switch/stay strategies and by different outcome valences were calculated and averaged across participants. The main effect of subsequent strategy in Figure 4 showed that the average ERSP displayed a transient increase during 250–500 ms (*p* < 0.01), mainly at the theta frequency band (the first two columns of Figure 4). Paired *t*-tests showed a significant ERSP difference during approximately 250–500 ms within 3–7 Hz; trials followed by a switch strategy displayed a stronger post-stimulus spectral power in this time-frequency region of interest (TF ROI) than those followed by a stay strategy (*p* < 0.001; the third column of Figure 4). It is also indicated by Figure 4 that the time-frequency characteristics computed directly using channel EEG data are homogeneous (i.e., with similar pattern), indicating a strong correlation between scalp EEG signals (representative midline sites of Fz, Cz, and Pz are shown in Figure 4). The interaction effect of outcome valence by subsequent strategy was not significant either based on the ERSP or on the ITC measurement (see Figure 5). For the sake of brevity, ERO images of the main effect of outcome valence and the interaction effect on ITC are provided in Appendix (see Figure A1 in Appendix, Part D).

### ICA results

**Figure 6:**
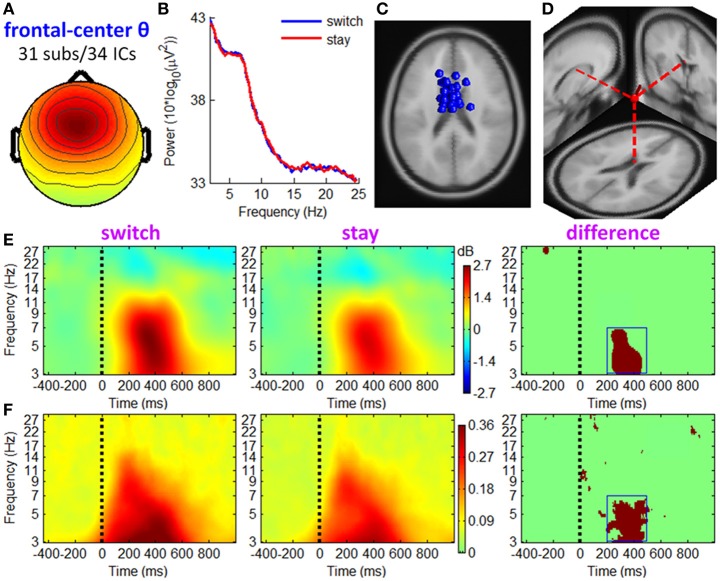
**IC properties of fronto-central theta cluster**. **(A)** Mean scalp maps across 34 ICs. **(B)** Mean power spectra. **(C)** Equivalent dipole locations of 34 ICs. **(D)** Mean dipole location and orientation, projected into a standard brain. **(E)** Mean ERSP image. Colors show significant deviations in log power (dB) from baseline; green indicates no significance (*p* > 0.01). **(F)** Mean ITC image. Colors show significant phase consistency across single-trial data (*p* < 0.01). Dark red regions in the third column of **(E,F)** show significant ERSP and ITC differences, respectively, between two conditions based on paired *t*-tests with a significance threshold of *p* < 0.001.

**Figure 7:**
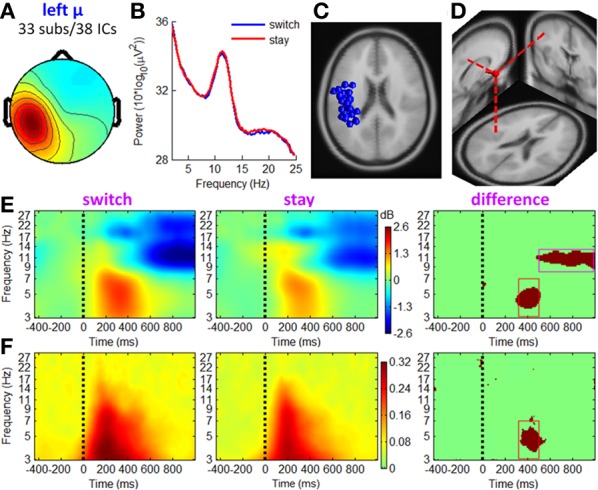
**IC properties of left mu rhythm cluster**. **(A)** Mean scalp maps across 38 ICs. **(B)** Mean power spectra. **(C)** Equivalent dipole locations of 38 ICs. **(D)** Mean dipole location and orientation. **(E)** Mean ERSP image. **(F)** Mean ITC image. The red and magenta boxes define TF ROIs for single-trial feature extraction.

**Figure 8:**
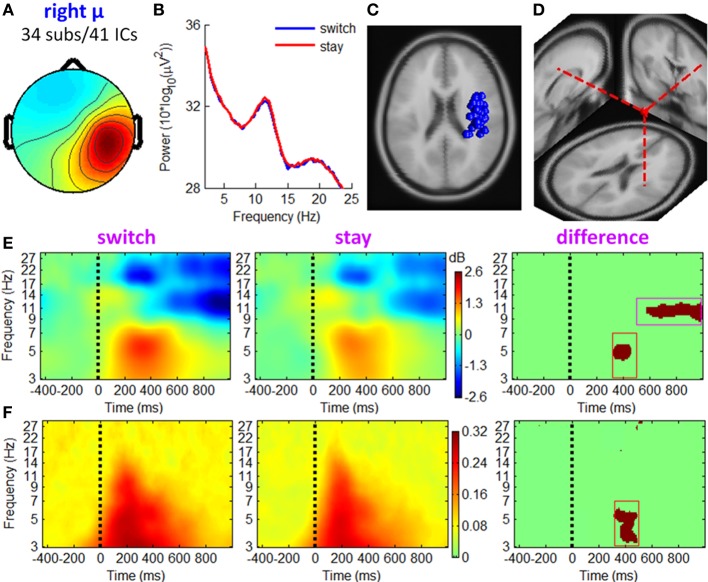
**IC properties of right mu rhythm cluster**. **(A)** Mean scalp maps across 41 ICs. **(B)** Mean power spectra. **(C)** Equivalent dipole locations of 41 ICs. **(D)** Mean dipole location and orientation. **(E)** Mean ERSP image. **(F)** Mean ITC image.

**Figure 9:**
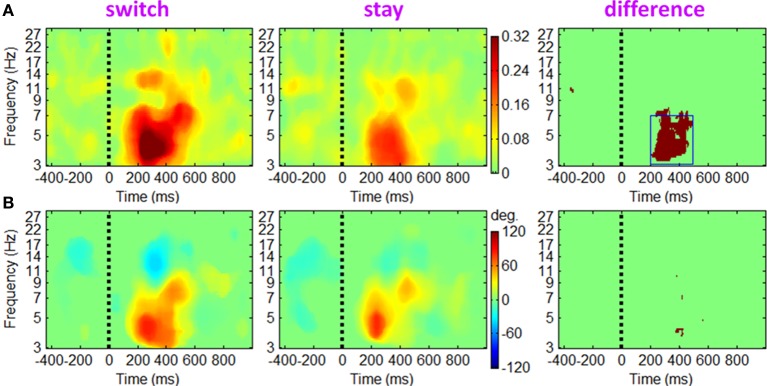
**The ERCOH measurements between fronto-central theta and the left mu IC clusters**. **(A)** The phase cross-coherence magnitude. Colors show significant ERCOH magnitude between the two IC clusters (*p* < 0.01). The dark red region in the third column shows significant ERCOH differences between the two conditions based on paired *t*-tests (*p* < 0.001). **(B)** This figure highlights the phase difference between the two IC clusters at time-frequency points where ERCOH magnitude in **(A)** is significant.

Among the acquired ten IC clusters, three of them contained ICs from less than 20 participants (i.e., insufficient to represent a common IC pattern across 39 participants) so they were not further analyzed in this study. The remaining seven clusters largely reproduced the IC clusters in previous studies (e.g., Makeig et al., 2002, 2004). Significant ERSP and ITC differences between switch and stay trials were found in three IC clusters, namely fronto-central theta cluster and left/right mu rhythm clusters. Together, these three IC clusters accounted for 74.8% of the variance of the grand-mean averaged ERP difference wave between switch and stay conditions at all channels in the 1 s time window following feedback onset (indicated by the EEGLAB function *std_envtopo*). Dynamic properties of these clusters are summarized in Figures 6–9.

#### Fronto-central theta cluster

Figure 6 shows the IC properties of fronto-central theta cluster, which included 34 ICs from 31 participants. The location of the equivalent dipole for a radially-oriented cortical source patch is typically deeper than the cortical patch itself (Baillet et al., 2001; Makeig et al., 2004). Consistently, the mean equivalent dipole location of fronto-central theta cluster (see Figure 6D) generally coincided with sources in or near the medial frontal cortex (MFC). ERSP images (see Figure 5E) indicate that theta spectral power increased significantly during 250–500 ms post-stimulus, with a larger increment in trials followed by switch than by stay strategies (see the dark red region in the right subplot of Figure 6E, paired *t*-test, *p* < 0.001). The ITC measurement in Figure 6F shows partial phase coherence across trials at approximately 3–7 Hz, occurring at 200–500 ms after outcome presentation. Compared with stay trials, switch trials had a higher degree of phase coherence of theta band oscillations that centered at this TF ROI (see the dark red region in the right subplot of Figure 6F, paired *t*-test, *p* < 0.001). Thus, the post-stimulus theta burst phenomenon comprised both a frequency-specific power increase and significant phase locking.

#### Mu rhythm clusters

Figures 7, 8 show the IC properties of left and right mu rhythm clusters. The mean equivalent dipoles are located roughly over the hand motor cortex. ERSP images (see Figures 7E, 8E) indicate that distinct spectral peaks near 10 and 20 Hz were strongly blocked from about 500 ms post-stimulus, with a larger decrement in trials followed by switch than those followed by stay strategies (TF ROI: 500 to 1000 ms × 8 to 13 Hz; paired *t*-test, *p* < 0.001).

In line with the fronto-central theta cluster, the mu rhythm clusters contained a theta pattern concurrent with a mean theta power increment at approximately 200–500 ms post-stimulus, which was consistent with Makeig et al. (2004). However, the ERSP differences between switch and stay trials were significant at a TF ROI of 320 to 500 ms × 3 to 7 Hz (paired *t*-test, *p* < 0.001). The ITC measurement in Figures 7F, 8F showed a partial phase coherence between outcome presentations and single trials at approximately 3–7 Hz, lasting from 320 to 500 ms post-stimulus; switch trials had a higher degree of phase coherence of EROs than stay trials at this TF ROI (paired *t*-test, *p* < 0.001).

#### ERCOH between fronto-central theta and mu ICs

To examine event-related changes in the coupling of IC activations between different brain regions, we analyzed the ERCOH measure between the activities of fronto-central theta and two mu IC clusters. Since the results derived from the left and right mu ICs were similar, only the ERCOH between fronto-central theta and left mu ICs is shown in Figure 9. The ERCOH amplitude in Figure 9A showed that significant theta phase coherence appeared in the data, indicating a transient post-stimulus phase linkage between the fronto-central and sensorimotor cortical regions. This phase cross-coherence was more prominent in the switch trials compared with stay trials at a TF ROI of 200 to 500 ms × 3 to 7 Hz (paired *t*-test, *p* < 0.001). Mean coherence phase lag between the two IC clusters suggests that the theta rhythm in fronto-central area led the theta rhythm in sensorimotor cortex with a phase offset about 60° (see Figure 9B).

#### Other IC clusters

**Figure 10:**
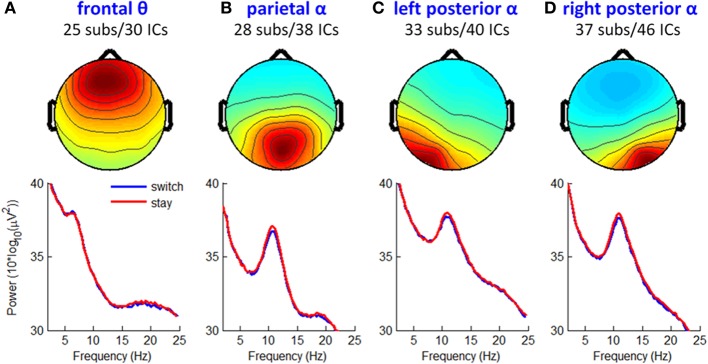
**Mean IC maps and power spectra of other four prominent IC clusters**. **(A)** Frontal theta rhythm cluster. **(B)** Parietal alpha rhythm cluster. **(C,D)** Left and right posterior alpha rhythm clusters.

Figure 10 shows the mean IC scalp maps and power spectra of the other four prominent IC clusters obtained in this study. Paired *t*-tests found no reliable and significant ERSP or ITC differences between switch and stay trials across individual ICs (*p* > 0.001).

### Single-trial prediction results

The AUC measurement among 39 participants was 0.65 ± 0.06. The AUC for the label-permuted distribution was calculated using permutation statistics for each participant [mean 99% CI = (0.50, 0.59), *SD* of 0.5% = 0.06, *SD* of 99% = 0.05]. Hence, the AUC given by the logistic regression classifier achieved a significance level of *p* < 0.01. In conclusion, single-trial classifier revealed that a satisfactory prediction of subsequent behavior could be achieved using ERP and ERO features elicited by current outcome presentations.

## Discussion

The current study investigated the potential relation between current outcomes and subsequent decision-making with behavioral and electrophysiological measures. The behavioral results indicated that participants were more prone to switch between high- and low-risk options after receiving positive outcomes. We suggest this finding reflected a fallacious belief about random events. In our opinion, many participants falsely believed that when continuously choosing the same option, they were less likely to receive identical outcomes in adjacent trials (i.e., they falsely believed that repeated trials were not statistically independent; see Tversky and Kahneman, 1971). Accordingly, choice-switching happened more often following positive outcomes than following other conditions.

The ERP results indicated that the P3 component was sensitive to future decisions, such that choice-switching was more likely to be associated with a larger P3 than the decision to stay on the same option (see also Zhang et al., 2013). In light of this finding, one might suggest that the larger P3 associated with choice-switching simply reflected that subsequent switch trials were more likely to have followed positive outcomes, which were also associated with a larger P3 (i.e., sampling bias). However, it is important to emphasize that, in contrast to the behavioral results, the effect of strategy was insensitive to outcome valence for the ERP data. Consequently, this alternate account of the P3 finding was considered relatively unlikely. The discrepancy has also been reported in our previous work and may indicate that the behavioral and ERP findings capture different aspects of the influence of current outcomes on future decisions (Zhang et al., 2013). Furthermore, the ERO results indicated that the fronto-central theta and left/right mu rhythms were also linked to the factor of subsequent strategy, such that increased spectral power and higher degrees of phase coherence were associated with switch trials compared to stay trials. Finally, single-trial analyses based on either channel- or ICA-features, or the combination of the two, revealed a satisfactory prediction of subsequent decision.

The current study found that the amplitude of the P3 following outcome presentation predicted choice-switching in subsequent trials, which was consistent with some previous studies (e.g., San Martín et al., 2013; Zhang et al., 2013), but contradicted other studies focused on the effect of the FRN rather than the P3 (e.g., Cohen and Ranganath, 2007; Cavanagh et al., 2010). In order to reconcile this contradiction, the importance of methodological variability be examined. The current study, as well as many others which discovered a relation between the P3 amplitude and future decisions (San Martín et al., 2013; Zhang et al., 2013), used a risk decision-making paradigm, of which the major characteristic is that the available options differ in levels of reward magnitude. In contrast, in those studies which highlighted the prediction role of the FRN, the magnitude of potential payoff was fixed such that outcome feedback only indicated the dimension of valence (Cohen and Ranganath, 2007; Cavanagh et al., 2010). Therefore, it is reasonable to hypothesize that the P3 functions as a predictor of future decisions when participants consider both outcome valence and magnitude in the current context, while the FRN plays the same role when only the valence is evaluated. Consistent with this explanation, the two-stage sequential model of outcome evaluation suggests an early, quick detection of the valence of an outcome (indexed by the FRN) and a late, deliberate integration of both valence and magnitude (indexed by the P3) (Wu and Zhou, 2009; Philiastides et al., 2010; see also Gu et al., 2011). In short, the pattern of the relationship between the electrophysiological responses to current outcomes and future behavior is highly context-sensitive and may depend on the features of outcome feedback.

The EROs in this study also provided important information about the electrophysiological mechanisms of the associations between current outcomes and future behavior. Specifically, the temporal location of ERP components and the ITC images suggested that theta oscillations make a sizable contribution to the P3 component, such that the P3 amplitude difference between “switch” and “stay” trials (peaked at approximately 400 ms, see Figure 3A) were most likely generated from enhanced intertrial phase coherence of theta oscillatory in switch condition than stay condition (see the dark-red region in right panels of Figures 6–8F; see also Cohen et al., 2009a; Nigbur et al., 2012). We make this inference based upon the fact that the ERP and ongoing EEG oscillations interact and relate to each other, reflecting different aspects of brain responses to an event, and that the post-stimulus ERP can mainly be accounted for by the (partial) phase locking or resetting of the EEG rhythms (Makeig et al., 2002; Klimesch et al., 2007). The ITC images, measuring the degree of phase resetting (i.e., phase consistency or phase locking) of EEG activity in single trials, revealed that during the P3 period, the uniform phase distribution across trials was replaced by a phase distribution weighted toward a dominant phase in the theta band. Moreover, this stimulus-locked ITC differed substantially between the switch and stay trials (Figures 6–8F). The finding that the theta phase coherence contributed to the P3 component is consistent with previous studies (Zervakis et al., 2011).

Furthermore, the theta oscillations located in the MFC (see Figure 6) and sensorimotor cortex (see Figures 7, 8) were significantly correlated with a subsequent decision-making strategy of switch and stay. In most previous studies, the cognitive function of fronto-central theta rhythm has been interpreted in terms of the model of reinforcement learning (e.g., Kamarajan et al., 2008, 2012; Marco-Pallares et al., 2008; Cohen et al., 2009a,b, 2012; Cavanagh et al., 2009). However, Cavanagh et al. (2010) recently reported that MFC theta power was not linked to the degree of learning from previous outcomes, but were reflective of a general operating mechanism involved in action monitoring and cognitive control (see also Cohen et al., 2009b; Cavanagh et al., 2012; Nigbur et al., 2012). In this study, while the chances of winning and losing were equal regardless of task performance (i.e., no optimal strategy could be learned), the fronto-central theta rhythms were still associated with subsequent decisions. Thereby, we agree with Cavanagh et al. (2010) that the MFC theta-band activity elicited by outcome feedback represents a general top-down process that is necessary for multiple forms of behavioral adaptation and strategic adjustment.

Another interesting finding is that the mu rhythm played an important role in predicting subsequent decision strategies. The AUC scores of single-trial classification (indicating the validity of the classifiers) based on the combined channel- and ICA-features were significantly higher than those only based on channel-features, mainly due to the ICA-derived mu oscillations in left and right sensorimotor cortices (see Figures 7, 8). The mu rhythm usually desynchronizes with imagery or actual motor movements (McFarland et al., 2000; Pfurtscheller et al., 2006). In the present study, the mu rhythm desynchronized more obviously before a switch decision than a stay decision, time-locked to the current outcome presentation (rather than motor responses). Thus, the observed mu rhythm phenomenon was associated with action planning and preparation rather than actual movements (for the relationship between mu rhythm and action planning, see Marshall and Meltzoff, 2011; Sabate et al., 2012). The rhythmic fluctuation of mu power might represent the accumulation of external information in the sensorimotor cortex, such that the motivational value of the current outcome is integrated in the process of subsequent action preparation (Wyart et al., 2012). This action-planning-related mu rhythm desynchronized as soon as the appearance of outcome feedback, indicating that the human brain may plan future decision-making at the stage of current outcome evaluation.

Furthermore, the results of ERCOH (see Figure 9) showed that the fronto-central theta significantly interacted with the theta oscillation in the sensorimotor cortex (see also van de Vijver et al., 2011: the frontal theta showed increased intersite phase synchrony with sensorimotor cortex). In our opinion, while the theta rhythm reflects a general process of cognitive control that underlies behavioral adaptation, a period of increased theta phase coherence between MFC and sensorimotor cortices might help with re-adjusting sensory and motor expectancies (Makeig et al., 2004). This idea is consistent with the finding that the theta activity is required for the formation of an accurate motor plan and is associated with enhancements of motor performance (Perfetti et al., 2011b; Nigbur et al., 2012). It is also in line with one of our recent studies, in which the ERP source analysis revealed that the sensorimotor cortex was activated during outcome presentation (Zhang et al., 2013). However, owing to the limited spatial accuracy of the EEG technique (Cook et al., 1998), further brain-imaging studies may be necessary to verify our results and help to provide more precise localization. In addition, although we found the fronto-central theta and sensorimotor theta oscillations interacted with each other in this study, the directed connectivity between these two components is still awaited to be examined by using straightforward approaches for causal interaction such as phase transfer entropy (Lobier et al., 2014) and Granger causality (Granger, 1988).

To sum up, this study has provided novel findings about the neural mechanism of risk decision-making with ERP and ERO measures. We found that the P3 component was reflective of the processes of strategic adjustment. The corresponding cortical oscillations were represented as the theta rhythm phase synchrony between fronto-central and sensorimotor cortices. Regarding the well-acknowledged association between the P3 and the motivational significance of outcome events (Nieuwenhuis et al., 2005; Wu and Zhou, 2009), we propose a mechanism of the influence of current outcomes on future decisions. Specifically, we propose that when the motivational significance of an outcome is prominent, the activated neural system of action monitoring (indexed by the fronto-central theta) compares the anticipated consequences of different strategies in the current context (Makeig et al., 2004). At the same time, if behavioral adjustment is determined to be more favorable, a “go” signal may occur in the motor cortex (indexed by the sensorimotor theta) and would be stored in the motor memory structure (indexed by the sensorimotor mu) (Perfetti et al., 2011a). Then this “go” signal in sensorimotor area may induce a strategy switching in the following decision-making process. Accordingly, changes in decision-making strategy would occur subsequently if the behavioral motivation elicited by this signal overcomes the predominant behavioral tendency (e.g., risk preference). The mechanism described above explains how and when the current outcome information affects future behavioral selections.

### Conflict of interest statement

The authors declare that the research was conducted in the absence of any commercial or financial relationships that could be construed as a potential conflict of interest.

## Figures and Tables

**Figure A2:**
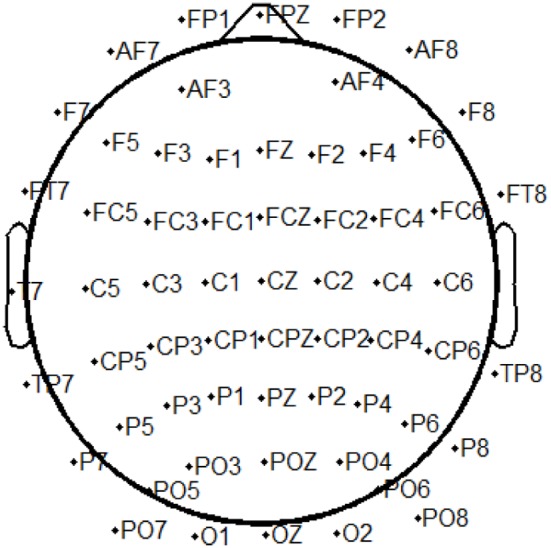
**Electrode positions and labels of the data set in the current study**.

## Appendix

### A. Instructions on the meanings of the symbols

In the current study, the participants were informed about the meaning of four possible outcome feedbacks before the task. The formal written instructions read as follows.

“In each trial, after you have chosen between 9 and 99, you will receive one of the four outcome feedbacks (i.e., positive, negative, neutral, and ambiguous outcomes). Positive outcomes (“+”) indicate that you have earned 9 or 99 bonus points according to your decision in this trial. Correspondingly, negative outcomes (“−”) indicate that you have lost 9 or 99 points from your total amount. Neutral outcomes (“0”) indicate that you have neither won nor lost any point in the current trial. Finally, ambiguous outcomes (“^*^”) are uninformative, which means the valence of the current outcome could be positive, negative, or neutral, but you have no knowledge about it.”

### B. Statistical analysis of the behavioral and ERP data

Statistical analyses were performed using SPSS Statistics 17.0 (IBM, Somers, USA). The results have been presented as mean ± standard deviation (SD). The significance level (α) was set at 0.05, unless specified otherwise. Greenhouse-Geisser correction for ANOVA tests was used whenever appropriate. The Bonferroni method was used to correct *p* values to address Type I error inflation. Significant interactions were analyzed using simple effects models. Partial eta-squared (η^2^_*p*_) values were reported to demonstrate the effect size of ANOVA tests, where 0.05 represents a small effect, 0.10 indicates a medium effect, and 0.20 represents a large effect (Cohen, 1973).

### C. Technique details of ICA

The epoched data of each participant were concatenated to form a 640 epochs × 61 channels matrix, followed by visual inspection to discard epochs containing irregular noise (e.g., non-stereotyped, unique artifacts such as high-amplitude or high-frequency noise and linear noise). Typical physiological artifacts such as eye blinks, lateral eye movements, heartbeat, and temporal muscle noises were kept in the data. The *runica* algorithm of EEGLAB was then performed on a subject-to-subject basis as an implementation of extended infomax ICA (Bell and Sejnowski, 1995; Makeig et al., 1996; Lee et al., 1999) to obtain 61 ICs from each of 39 datasets. Default *runica* training parameters were used (stopping *W* matrix change = 10^−7^; maximum iteration number = 512). The method of using two rounds of ICA is suggested by many researchers, including the authors of EEGLAB, since the results of the initial round are informative in rejecting more trials (Meltzer et al., 2007; Miyakoshi et al., 2010; de Borst et al., 2012). Therefore, the ICA resulting data were further visually inspected to exclude remaining “bad trials” that containing a relatively large number of noisy IC activations. Afterwards, a second ICA was run on the pruned data, followed by the same epoch rejection procedure.

### D. ERSP and ITC analyses

The ERSP contains the narrow-band event-related desynchronization and synchronization and illustrates mean stimulus-locked EEG power deviations from baseline-mean power in decibels (dB) across a broad frequency range (Makeig, 1993). The ITC (first introduced by Tallon-Baudry et al., 1996, which termed the “phase locking factor”) measures the trial-to-trial phase consistency of EEG data at a particular latency and frequency; a value of 0 represents absence of synchronization between EEG data and the occurrence of experimental events; a value near 1 indicates perfect synchronization (i.e., near identical phase across trials at a given latency). These two time-frequency measurements were calculated using a Morlet wavelet with the number of cycles linearly rising from a minimum of 3 cycles at 3 Hz to a maximum of 7 cycles at 35 Hz within one analysis window. This modified wavelet transform was selected to optimize the trade-off between temporal resolution at lower frequencies and stability at higher frequencies. Log-spaced 80 frequencies ranging from 3 to 35 Hz were calculated from 1 s prior to and ending up to 2 s following the onset of outcome presentation with baseline correction from −1 to 0 s. When plotting the ERSP and ITC results across participants, two-tailed permutation statistics were computed with α set to 0.01 (extracting at random from baseline data and applied 200 times). To estimate the reliability of the ERSP and ITC differences between different conditions across 39 participants, paired parametric statistics with Bonferroni correction were performed at each pixel with α set to 0.001 (Makeig et al., 2004; Onton et al., 2005). Common ERSP baseline was used in all conditions.

The ERSP images of the main effect of outcome valence (see Figure A1) are provided as follows. ITC plots of main effects are omitted because there was no reliable and significant phase coherence difference between conditions.

### E. IC cluster procedure

In this study, the IC feature vectors for clustering were composed of power spectra with dimensions reduced to 3 principal components (PCs) by principal component analysis (PCA), averaged ERPs (reduced to 4 PCs), equivalent dipole locations (dimension = 3), scalp map gradients (reduced to 5 PCs), averaged ERSPs (reduced to 5 PCs), and ITCs (reduced to 5 PCs). The equivalent dipole location measure was multiplicatively weighted by a factor of 2; other measures were given a weight of 1, and all features were normalized. Finally, the 25-dimensional IC measure was compressed again by PCA into a 12-dimensional cluster vector. The derived 380 ICs from the 39 participants were clustered by applying the *k*-means algorithm based on the 12-dimensional measure, resulting in ten mutually exclusive IC clusters by minimizing the variability within and maximizing the variability between clusters. ICs with a distance larger than two SDs from the mean of any cluster centroid were excluded as outliers.

### F. Power spectra and ERCOH analyses

Component power spectra were calculated by averaging fast Fourier transform spectra from 0 to 800 ms post-stimulus (window length = 256 points, overlap = 128 points).

An event-related cross-coherence (ERCOH) measure embedded in EEGLAB was employed to estimate the degree of phase synchronization between the activations of two IC clusters. The magnitude of ERCOH varies between 0 (i.e., a complete absence of synchronization) and 1 (i.e., perfect synchronization) while the phase of ERCOH indicates the potential causal relationship between two IC clusters (under the minimum phase assumption that the actual phase lag is less than ±180°) (Delorme and Makeig, 2004).

### G. Electrode locations

In addition to electrooculogram (EOG) and referential electrodes, 61 channels were used for EEG data collection. The electrode locations are illustrated in Figure A2.
